# Echocardiographic effects of sodium-glucose cotransporter 2 inhibitors in single ventricle circulatory failure

**DOI:** 10.1016/j.ijcchd.2025.100603

**Published:** 2025-06-21

**Authors:** Ralph M.L. Neijenhuis, Madelien V. Regeer, Niki L. Walker, Amanda Hunter, Philippine Kiès, Eduard R. Holman, J. Wouter Jukema, Monique R.M. Jongbloed, Gruschen R. Veldtman, Anastasia D. Egorova

**Affiliations:** a CAHAL, Center for Congenital Heart Disease Amsterdam-Leiden, Leiden University Medical Center, Leiden, the Netherlands; b Department of Cardiology, Leiden University Medical Center, Leiden, the Netherlands; c Scottish Adult Congenital Cardiac Service (SACCS), Golden Jubilee University National Hospital, Glasgow, United Kingdom; d Netherlands Heart Institute, Utrecht, the Netherlands; e Department of Anatomy & Embryology, Leiden University Medical Center, Leiden, the Netherlands; f Adult Congenital Heart Disease Center, Helen DeVos Children's Hospital, Grand Rapids, MI, United States of America

**Keywords:** Fontan, Univentricular, Single ventricle circulatory failure, Congenital heart disease, Echocardiography, Sodium-glucose cotransporter 2 inhibitors, Heart failure

## Abstract

**Background:**

Single ventricle patients are at high risk of developing circulatory failure. There is limited evidence for pharmacological treatment. This study assessed the echocardiographic changes in ventricular function during sodium-glucose cotransporter 2 inhibitor (SGLT2i) therapy in patients with single ventricle failure (SVF).

**Methods:**

SVF patients with a baseline transthoracic echocardiogram within six months before starting SGLT2i and at least one echocardiographic examination within twelve months follow-up were included from a real-world international registry of adult congenital heart disease patients on SGLT2i. Mixed models were used to evaluate longitudinal changes in ventricular function and differences between patients with SVF with ≥ moderately reduced systolic function (SVFrEF) and with ≤ mildly reduced function (SVFpEF).

**Results:**

Thirteen patients were included. The median age was 21 [20–42] years, 8 (61.5 %) were female, 10 (76.9 %) had a Fontan circulation, 8 (61.5 %) had SVFrEF, and 5 (38.5 %) SVFpEF at the start of SGLT2i. The mean follow-up was 7.6 ± 3.3 months. End-systolic area decreased significantly in all patients (−1.6 cm^2^ per month, p = 0.007) in the first 100 days. Fractional area change improved in the first 100 days in SVFrEF patients (3.5 %-point per month, p < 0.001), while SVFpEF patients remained stable. There was a significant improvement in the free wall strain in all patients (−0.3 %-point per month, p = 0.036) but not in global longitudinal strain (p = 0.087). Isovolumic acceleration also improved in the first 100 days (0.5 m/s^2^ per month, p = 0.010).

**Conclusions:**

Echocardiographic signals of improved ventricular function were observed in the first year of SGLT2i therapy in patients with SVF.

## Highlights

• The effects of SGLT2i in single ventricle failure (SVF) are unknown.
• Mixed models were utilized for real-world repeated measurement analyses.
• Although cautious, there are signs of improved ventricular function after SGLT2i.
• SVF patients with reduced systolic function might benefit more from SGLT2i.
• Future studies should evaluate SVF phenotype-specific responses to SGLT2i.

## Graphical abstract

**Figure:**
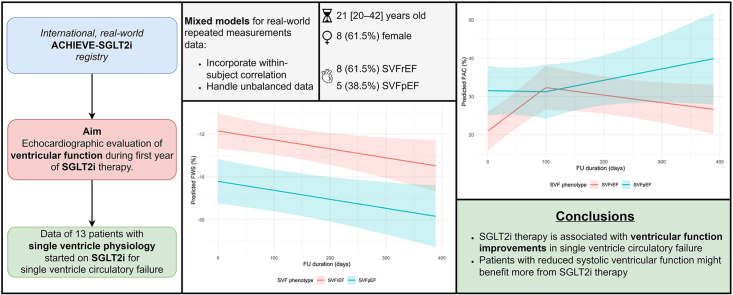

## Abbreviations list

ACHD: Adult congenital heart disease
AVV: Atrioventricular valve
FAC: Fractional area change
FWS: Free wall strain
GLS: Global longitudinal strain
IVA: Isovolumic acceleration
SGLT2i: Sodium-glucose cotransporter 2 inhibitor(s)
SVFpEF: Single ventricle failure with preserved ejection fraction
SVFrEF: Single ventricle failure with reduced ejection fraction
TAPSE/MAPSE: Tricuspid/mitral annular plane systolic excursion
TTE: Transthoracic echocardiography

## 1 Introduction

Patients with single ventricle physiology represent one of the most complicated hemodynamic subtypes within adult congenital heart disease (ACHD). In this heterogeneous population, most patients are able to undergo surgical palliation with the Fontan procedure, resulting in a circulation in which the single functional ventricle supports the systemic and pulmonary circulation ‘in series’. Single ventricle patients who do not undergo Fontan palliation may have an intrinsically ‘balanced circulation’, in which pulmonary blood flow is not too excessive nor too little. Alternatively, single ventricle patients may require systemic-to-pulmonary artery shunts that augment pulmonary blood flow, which in turn predisposes them to the development of pulmonary overcirculation and pulmonary vascular disease [1]. Ultimately, single ventricle physiology patients are prone to develop heart failure at a significantly higher rate than other congenital heart disease groups. Fontan failure occurs in over 30 % of patients across all ages, and prevalence increases to nearly 100 % in patients over 50 years old [2]. Heart failure is the principal cause of death in the Fontan population, and the overall adult survival rate is 90 % at 30 years of age, 80 % at 40, and 61 % at age 50 [3].

Despite this high morbidity and mortality burden, the current ACHD guidelines provide few recommendations for the management of single ventricle circulatory failure and substantiate this key knowledge gap as being due to a poor understanding of the underlying failure mechanisms and the absence of evidence-based treatment options [1,4]. The guidelines do emphasize that pharmacotherapy should be initiated cautiously given the delicate balance between ventricular preload and systemic afterload.

In recent years, sodium-glucose cotransporter 2 inhibitors (SGLT2i) have emerged as a cornerstone in managing conventional heart failure [5,6]. Although SGLT2i appear safe and well-tolerated in the ACHD population, there have been few small studies that specifically evaluated SGLT2i in the single ventricle population or included single ventricle patients, and potential beneficial effects remain to be elucidated [[7], [8], [9], [10], [11], [12], [13], [14]]. Systolic and diastolic ventricular dysfunction are the most prevalent causes of single ventricle circulatory failure [15]. Due to the proposed pleiotropic mechanisms of action, SGLT2i are a promising treatment option for single ventricle circulatory failure, and their effect on ventricular function deserves further evaluation [16]. This study aimed to delineate comprehensively the longitudinal echocardiographic changes in ventricular function observed during the first year of SGLT2i therapy in patients with single ventricle circulatory failure.

## 2 Methods

### 2.1 Study design

Patients with a physiological single ventricle circulation included in the international, real-world ACHIEVE-SGLT2i (**A**dult **C**ongenital **H**eart disease **I**nternational **EV**aluation of the **E**ffectiveness of **SGLT2i**) registry (National Clinical Trial number: NCT06932081) were assessed for eligibility for this retrospective on-treatment study. Patients with single ventricle physiology (≥18 years of age) had to be initiated on an SGLT2i for symptomatic circulatory failure and had to have a baseline transthoracic echocardiogram (TTE) within six months before starting SGLT2i and at least one TTE in the outpatient setting between six weeks and twelve months follow-up available for offline analysis.

Single ventricle circulatory failure was adjudicated by the treating cardiologists who initiated SGLT2i, based on an extrapolation of the universal definition of heart failure to the complex single ventricle hemodynamics [17]. Patients with heart failure symptoms and ≥ moderately reduced systolic systemic ventricular function were classified as having single ventricle failure with a reduced ejection fraction (SVFrEF), and patients with ≤ mildly reduced systolic ventricular function as single ventricle failure with a preserved ejection fraction (SVFpEF), in line with previous literature [8].

### 2.2 Ethics

Appropriate medical ethical board approval was obtained at the participating centers (Leiden University Medical Center Medical Research Involving Human Subjects Act [WMO] committee division 1 protocol reference 2022-068, Southwest – Central Bristol Research Ethics Committee reference 24/SW/0036). The study was conducted in accordance with the ethical standards of the institutional and/or national research committees, 2013 Declaration of Helsinki, and in line with the STrengthening the Reporting of OBservational studies in Epidemiology (STROBE) statement [18].

### 2.3 Data collection and TTE assessment

**Fig. 1:**
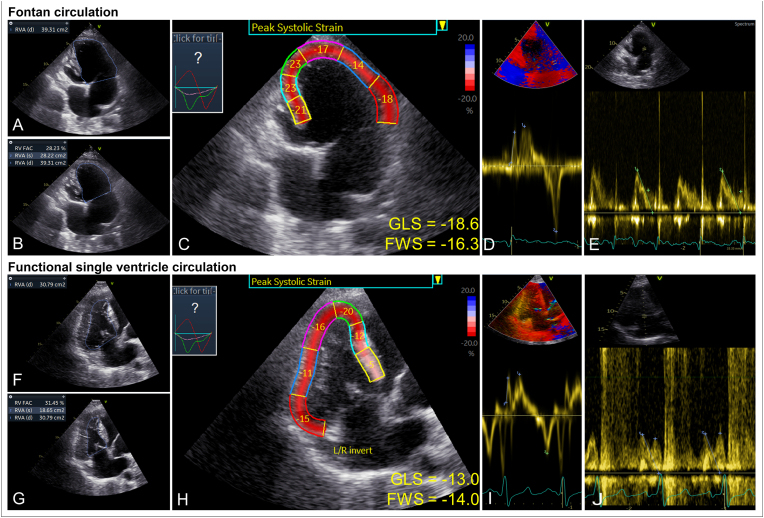
**Title.** TTE assessment methodology Fig. 1**Caption.** The top part of figure (A–E) shows the ventricular function assessment in a patient with tricuspid atresia and a left ventricle dominant Fontan circulation, the bottom part (F–J) in a patient with congenitally corrected transposition of the great arteries, double-outlet right ventricle, subaortic ventricular septal defect, and pulmonary atresia, palliated with a Glenn shunt and Blalock-Thomas-Taussig shunts. A&F) end-diastolic area (cm^2^), B&G) end-systolic area (cm^2^) and FAC calculation, C&H) strain analysis including GLS, segmental strain values, and calculation of the FWS by averaging the 3 lateral strain segments, D&I) tissue Doppler imaging measurements S’ (measurement 1, m/s), e’ (measurement 2, m/s), and IVA (slope measurement 3, m/s^2^), E&J) E and A velocities (m/s) measured on (at least) two complexes on pulsed-wave Doppler. *FAC, fractional area change; FWS, free wall strain; GLS, global longitudinal strain; IVA, isovolumic acceleration; RV, right ventricle; RVA, right ventricle area; TTE, transthoracic echocardiography.*

All clinical data were retrieved from the electronic health records. All TTEs obtained in an outpatient clinic setting as part of routine care, performed at variable time points, were included. Data was collected until: 1) 12 months follow-up, 2) the last date of data inclusion (if occurring at less than 12 months follow-up), 3) permanent discontinuation of SGLT2i therapy, 4) any catheter or surgical intervention influencing the patient's hemodynamic status, 5) loss to follow-up, or 6) death. R.M.L.N. was blinded to the patient's clinical status when performing the offline analyses, and supervised by two experienced European Association of Cardiovascular Imaging (EACVI) certified ACHD imaging cardiologists (M.V.R., G.R.V.). Each case was reviewed jointly by R.M.L.N., M.V.R., and G.R.V. before analysis to reach a consensus on the relevant anatomical landmarks and image interpretation, enhancing internal consistency. A dedicated TTE evaluation workflow was adapted to the single ventricle population [19]. The following parameters were assessed: systemic ventricle end-diastolic diameter (mm), apex-base length (mm), mid diameter (mm), free wall thickness (mm), end-diastolic area (cm^2^), end-systolic area (cm^2^), fractional area change (FAC, %), tricuspid/mitral annular plane systolic excursion (TAPSE/MAPSE, mm), systemic ventricle global longitudinal strain (GLS, %), free wall strain (FWS, %), separate segmental strain (%), and atrioventricular valve (AVV) regurgitation grades. Additionally, the tissue Doppler imaging parameters isovolumic acceleration (IVA, m/s^2^), S’ (m/s), and e’ (m/s) were measured at the lateral wall adjacent to the dominant AVV. E and A velocities (m/s) were measured over the dominant AVV. Strain measurements were performed using Q-analysis in EchoPAC™ (GE Healthcare, Chicago, IL, USA). The endocardial border was traced manually in the apical four-chamber view, including the interventricular septum if present and when contributing to the systolic function. Systolic systemic ventricle function was additionally classified into four qualitative categories based on a combination of GLS, FAC, and visual assessment. A detailed depiction of the ventricular function assessment is presented in Fig. 1.

### 2.4 Statistical analysis

The statistical analyses were performed with SPSS v25 (IBM Corp, Armonk, NY, USA) and R Statistical Software (v4.4.2; R Core Team 2023). Normally distributed continuous variables were presented as mean ± standard deviation, non-normally distributed continuous variables as median [interquartile range], and categorical variables as frequencies with percentages in parentheses. The "nlme" package (v3.1.167; R Core Team 2024) was used to construct linear mixed models with various specifications to assess the longitudinal relationship between TTE parameters and time, modeled as a continuous variable in days, as previously published [20]. To enhance clinical interpretability, time-dependent coefficients were expressed as changes per month. Differential responses during follow-up between SVFrEF and SVFpEF patients were investigated by including the failure phenotype as a binary covariate. Random intercepts and the addition of random slopes were explored. To model potential non-linear changes, piecewise linear splines models were tested. Various correlation and variance structures were also assessed. Model selection was based primarily on the Bayesian information criterion (BIC), with secondary consideration of the Akaike information criterion (AIC) or likelihood ratio tests when appropriate. Model parsimony was prioritized over predictive capacity in the model selection process, and fit was verified through residual analysis. The predicted fixed effects were visualized with corresponding 95 % confidence intervals for fixed-effect variance. Statistical significance was defined as a two-sided p-value ≤0.05.

## 3 Results

### 3.1 Baseline characteristics

Thirteen patients with single ventricle circulatory failure initiated on SGLT2i therapy between December 2021 and October 2023 were included. The median age was 21 [20–42] years old, 8 (61.5 %) were female, 10 (76.9 %) had a Fontan circulation, 8 (61.5 %) had SVFrEF, and 5 (38.5 %) SVFpEF. Dapagliflozin 10 mg once daily was started in 9 (69.2 %) patients, and empagliflozin 10 mg once daily in 4 (30.8 %). The mean echocardiographic follow-up duration was 7.6 ± 3.3 months and 35 TTEs were included for analysis. All patients had a baseline TTE assessment, 6 patients had 1 follow-up TTE, 5 patients had 2, and 2 patients had 3. Detailed baseline characteristics and TTE parameters are presented in Table 1, Table 2, and individual anatomical and echocardiographic characteristics in Supplementary Table 1.

**Table 1:** Baseline characteristics.

	n = 13
Age, y	21 [20–42]
Sex, female	8 (61.5)

*Single ventricle anatomy*	
Fontan	
RV dominant (hypoplastic left heart syndrome)	5 (38.5)
LV dominant	4 (30.8)
Mixed/indeterminate ventricle	1 (7.7)
Other (double-outlet right ventricle + ventricular septal defect ± other lesions)	3 (23.1)

*Fontan type*	
Extracardiac Fontan	8 (61.5)
Lateral tunnel Fontan	2 (15.4)

*Single ventricle failure phenotype*	
SVFrEF	8 (61.5)
SVFpEF	5 (38.5)

Protein-losing enteropathy	1 (7.7)
Plastic bronchitis	0
Type 2 diabetes mellitus	0
Number of cardiac surgeries	3 [3–4]
Pacemaker	4 (30.8)

*Pharmacotherapy*	
MRA	11 (84.6)
ACEi/ARB/ARNI	9 (69.2)
Beta-blocker	8 (61.5)
Diuretic	8 (61.5)
PDE5 inhibitor	0

*Clinical parameters*	
Body surface area, m^2^	1.75 [1.46–1.79]
Heart rate, bpm	73 ± 8
Heart rhythm	
Sinus rhythm	10 (76.9)
Atrial rhythm	1 (7.7)
Atrial pacing	1 (7.7)
Ventricular pacing	1 (7.7)
Systolic blood pressure, mmHg	111 ± 14
Diastolic blood pressure, mmHg	67 ± 7

Values are n (%), mean ± standard deviation, or median [Q1 – Q3]. ACEi, angiotensin-converting enzyme inhibitor; ARB, angiotensin receptor blocker; ARNI, angiotensin receptor-neprilysin inhibitor; LV, left ventricle; MRA, mineralocorticoid receptor antagonist; PDE5, phosphodiesterase type 5; RV, right ventricle; SGLT2i, sodium-glucose cotransporter 2 inhibitor; SVFrEF/SVFpEF, single ventricle failure with reduced/preserved ejection fraction.

**Table 2:** TTE parameters at baseline.

	n = 13
*Systemic ventricle dimensions*	
End-diastolic diameter, mm	61 ± 17
Apex base length, mm	75 ± 8
Mid diameter, mm	54 ± 9
Free wall thickness, mm	9 ± 2
End-diastolic area, cm^2^	38.1 ± 6.6
End-systolic area, cm^2^	28.8 ± 6.9

*Global systolic ventricular function*	
Good	3 (23.1)
Mildly reduced	2 (15.4)
Moderately reduced	5 (38.5)
Severely reduced	3 (23.1)

*Dominant AVV regurgitation*	
Grade 1 or less	2 (15.4)
Grade 2	2 (15.4)
Grade 3	9 (69.2)
Grade 4	0

*Systolic function parameters*	
GLS, % (n = 12)	−13.9 ± 3.3
FWS, % (n = 12)	−13.3 ± 4.2
FAC, %	24.8 ± 9.1
TAPSE/MAPSE, mm (n = 12)	11.1 ± 1.8
S’, cm/s (n = 8)	6.0 ± 0.8
IVA, m/s^2^ (n = 8)	1.9 ± 0.9

*Diastolic function parameters*	
E/A ratio (n = 9)	1.7 ± 0.6
E/e’ ratio (n = 7)	6.3 [5.5–11.7]

Values are n (%), mean ± standard deviation, or median [Q1 – Q3]. Data is available for all patients unless specified otherwise. *AVV, atrioventricular valve; FAC, fractional area change; FWS, free wall strain; GLS, global longitudinal strain; IVA, isovolumic acceleration; TAPSE/MAPSE, tricuspid/mitral annular plane systolic excursion; TTE, transthoracic echocardiography*.

### 3.2 Ventricular remodeling

**Fig. 2:**
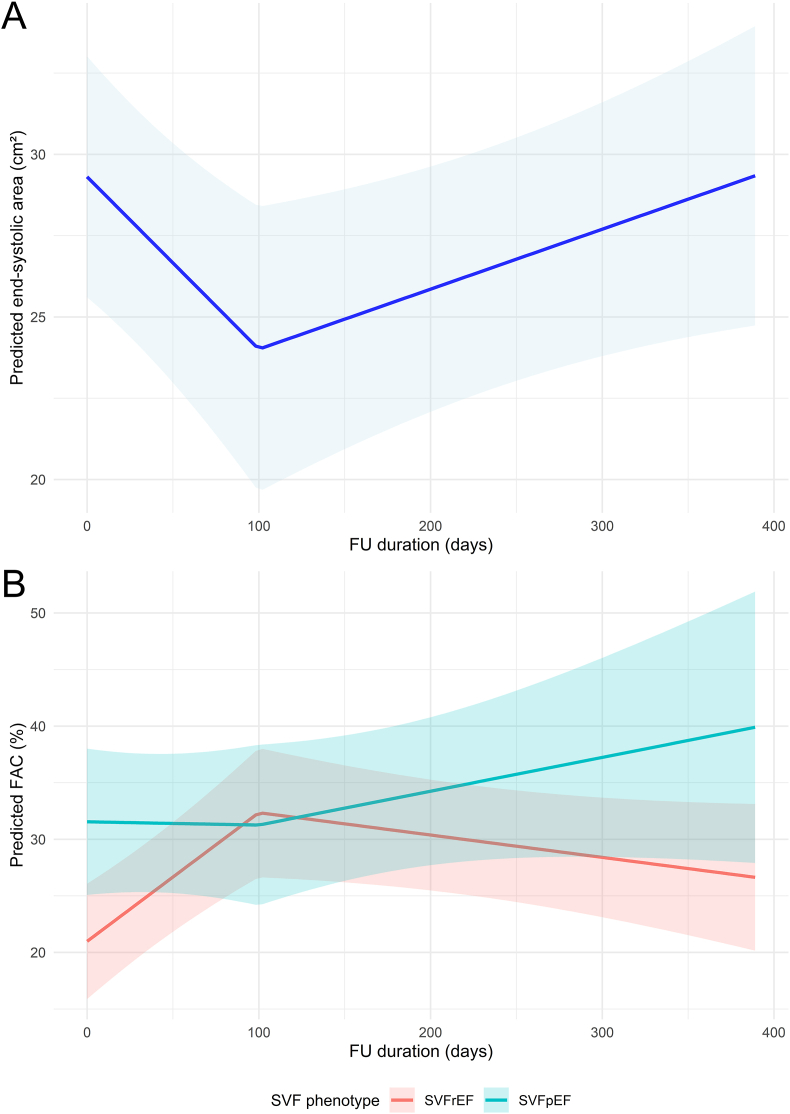
**Title.** Changes in end-systolic area and FAC after starting SGLT2i Fig. 2**Caption.** A) End-systolic area decreased significantly in the first 100 days after starting SGLT2i (−1.6 cm^2^ per month, p = 0.007), after which there was an increasing trend (0.6 cm^2^ per month, p = 0.051). B) Patients with SVFrEF had a significant improvement in FAC in the first 100 days (3.5 %-point per month, p < 0.001), after which the change was not significant (p = 0.141). SVFpEF patients had a significantly different trajectory from the SVFrEF patients and remained stable in the first 100 days (−0.1 %-point per month, interaction p = 0.007), after which the change was no longer significantly different between groups (interaction p = 0.081). *FAC, fractional area change; FU, follow-up, SGLT2i, sodium-glucose cotransporter 2 inhibitor; SVF, single ventricle failure, SVFrEF/SVFpEF, single ventricle failure with reduced/preserved ejection fraction*.

No temporal changes were observed in end-diastolic diameter (p = 0.682), apex-base length (≤100 days; p = 0.062, >100 days; p = 0.067), mid diameter (p = 0.846), free wall thickness (p = 0.087), or end-diastolic area (≤100 days; p = 0.323, >100 days; p = 0.071) after initiation of SGLT2i therapy. End-systolic area decreased significantly in the first 100 days of treatment (−1.6 cm^2^ per month, p = 0.007), after which there was a non-significant increasing trend (0.6 cm^2^ per month, p = 0.051) (Fig. 2A).

### 3.3 Systolic ventricular function

SVFrEF patients had a significant improvement in FAC in the first 100 days (3.5 %-point per month, p < 0.001), after which FAC remained stable (p = 0.141). SVFpEF patients remained stable in the first 100 days, which differed significantly from the improvement observed in the SVFrEF patients (−0.1 %-point per month, interaction p = 0.007). After 100 days, there were no significant changes between SVFrEF and SVFpEF patients (p = 0.081). The predicted changes in FAC are presented in Fig. 2B. No temporal changes in TAPSE/MAPSE were observed. SVFpEF patients did have significantly higher overall TAPSE/MAPSE values than SVFrEF patients (2.7 mm, p = 0.010) (Supplementary Fig. 1).

**Fig. 3:**
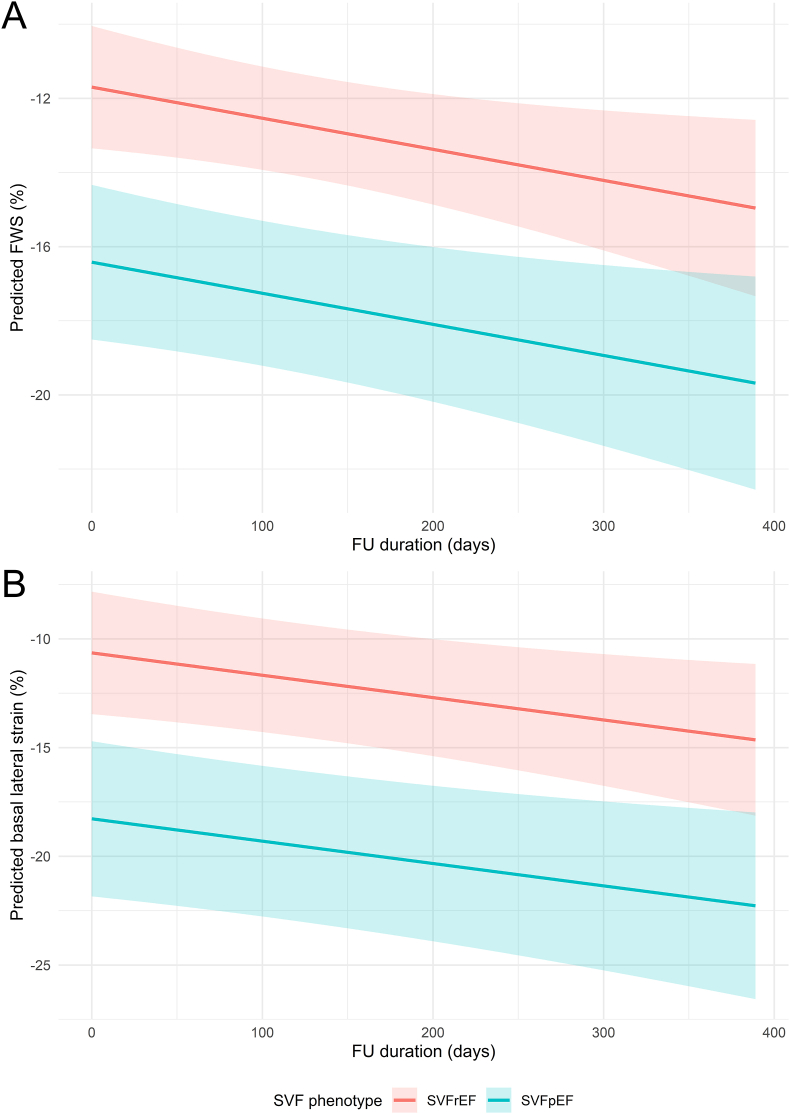
**Title.** Changes in FWS and basal lateral strain after starting SGLT2i Fig. 3**Legend.** A) Predicted longitudinal changes in FWS (%) after starting SGLT2i. There was a significant improvement in FWS over time (−0.3 %-point per month, p = 0.036). SVFpEF patients had significantly better strain values (4.7 %-point lower than SVFrEF patients, p = 0.003). B) Predicted longitudinal changes in basal lateral segmental strain (%). There was a significant improvement over time (−0.3 %-point per month, p = 0.032), and SVFpEF patients also had significantly better basal lateral segmental strain (7.6 %-point lower than SVFrEF patients, p = 0.006). *FU, follow-up; FWS, free wall strain; SGLT2i, sodium-glucose cotransporter 2 inhibitor; SVF, single ventricle failure, SVFrEF/SVFpEF, single ventricle failure with reduced/preserved ejection fraction*.

Although there was no significant overall improvement in GLS after starting SGLT2i (−0.1 %-point per month, p = 0.087), there was a significant improvement in FWS (−0.3 %-point per month, p = 0.036) and basal lateral segmental strain over time (−0.3 %-point per month, p = 0.032) (Fig. 3). All other strain segments demonstrated a trend towards improvement, but none were statistically significant. Compared to SVFrEF patients, SVFpEF patients had a significantly better overall GLS (−5.3 %-point, p < 0.001), FWS (−4.7 %-point, p = 0.003), mid septal segmental strain (−5.0 %-point, p = 0.018), mid lateral segmental strain (−4.9 %-point, p = 0.026), and basal lateral segmental strain (−7.6 %-point, p = 0.006) (Supplementary Fig. 2–4). The temporal response in strain measurements to SGLT2i showed no significant differences for the single ventricle failure phenotypes.

**Fig. 4:**
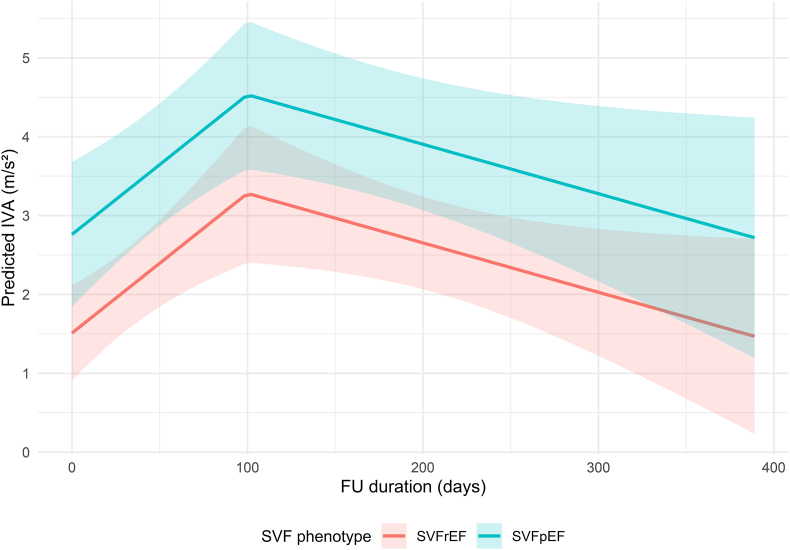
**Title.** Changes in IVA after starting SGLT2i Fig. 4**Legend.** There was a significant improvement in IVA in the first 100 days after starting SGLT2i (0.5 m/s^2^ per month, p = 0.010), after which there was a decreasing trend (−0.2 m/s^2^ per month, p = 0.075). SVFrEF patients had a significantly lower overall IVA than SVFpEF patients (1.3 cm^2^, p = 0.027). *FU, follow-up; IVA, isovolumic acceleration; SGLT2i, sodium-glucose cotransporter 2 inhibitor; SVF, single ventricle failure, SVFrEF/SVFpEF, single ventricle failure with reduced/preserved ejection fraction*.

IVA improved significantly in the first 100 days after starting SGLT2i (0.5 m/s^2^ per month, p = 0.010), and remained stable afterwards (p = 0.075). SVFrEF patients had a significantly lower overall IVA than SVFpEF patients (1.3 m/s^2^, p = 0.027) (Fig. 4). There were no temporal changes in S′, E/A ratio or E/e’ ratio after starting SGLT2i (p = 0.889, p = 0.626, and p = 0.289 respectively). The individual changes of all parameters with significant temporal changes after starting SGLT2i are included in Supplementary Fig. 5. All mixed model outputs and characteristics are presented in Supplementary Tables 2 and 3

## 4 Discussion

The main findings of this study are that in the first year of SGLT2i therapy for single ventricle circulatory failure, 1) there was a significant decrease in the end-systolic area of the systemic ventricle in all patients and a significant improvement in FAC in SVFrEF patients in the first 100 days, 2) there was a significant improvement in FWS and basal lateral segmental strain in all patients over time, although the GLS did not improve, and 3) there was a significant improvement in IVA in all patients in the first 100 days after SGLT2i initiation.

### 4.1 Phenotyping single ventricle circulatory failure

Single ventricle circulatory failure was categorized as either SVFrEF or SVFpEF. This dichotomization has been utilized previously, including in another recent study evaluating the use of SGLT2i in 14 Fontan patients [8,21]. *Konduri* et al. observed a reduction in NT-proBNP in 5 patients with at least moderately reduced ventricular function and concluded that SGLT2i might benefit Fontan patients with reduced ventricular function [8]. The current study focused on echocardiographic analyses, and clinical stratification of patients as SVFrEF or SVFpEF correlated with significant differences in multiple systolic function parameters. Moreover, an early improvement in FAC was only observed in SVFrEF patients, supporting the notion that SGLT2i may be of particular benefit in patients with reduced systolic ventricular function in the context of single ventricle circulatory failure.

Although systolic dysfunction is the most prevalent cause of single ventricle circulatory failure, the chronic state of hemodynamic stress in the single ventricle population does not always lead to contractile dysfunction, and it is imperative to recognize that a dichotomous categorization of single ventricle circulatory failure is an oversimplification [[22], [23], [24]]. Moreover, the observed discordance between GLS, FAC, and visual function assessment in some patients highlights the complexity of categorizing ventricular dysfunction. *Van Puyvelde* et al. identified five distinct and often co-existing mechanisms of Fontan failure: systolic ventricular dysfunction, AVV regurgitation, high pulmonary vascular resistance, restrictive physiology, and pathway obstruction [23]. Due to the lack of availability of invasive hemodynamic data and limited sample size, it was not possible to evaluate the changes after starting SGLT2i for each of these etiologies in the current cohort. Future research should aim to assess the treatment response in these different phenotypes, as SGLT2i might have deleterious effects on cardiac output in single ventricle circulation patients who are particularly preload-dependent, such as those with obstruction or high pulmonary vascular resistance physiologies [10].

### 4.2 TTE in single ventricle patients

Imaging of single ventricle patients is challenging. Due to the heterogeneity in ventricular geometry and individual anatomy, the imaging protocol for Fontan patients proposed by the International Society of Adult Congenital Heart Disease (ISACHD) used as reference for this study regards FAC as the most reliable marker of systolic function, as it makes no assumptions about ventricular geometry [19]. Additionally, FAC has been shown to correlate well with invasive pressure-volume loop indices of systolic function [25]. While the ISACHD protocol does not discuss the value of strain analysis, IVA is mentioned as a potentially interesting parameter that does not rely on ventricular geometry. However, no robust correlation between IVA and invasive pressure-volume loops could be established [19,25]. Moreover, there appear to be only two other studies that evaluated IVA in the single ventricle population [26,27]. So, while an overall improvement in IVA was observed after starting SGLT2i, and it appears to be an appealing geometry- and load-independent surrogate of myocardial contraction, better validation of IVA is required in the single ventricle population. The value of more conventional echocardiographic systolic function parameters (including strain) as independent predictors of adverse clinical outcomes has been demonstrated, and TTE remains a valuable tool for monitoring longitudinal changes in ventricular function in this cohort [21,28].

### 4.3 SGLT2i in the single ventricle population

To our knowledge, there have been four studies specifically focusing on the use of SGLT2i in single ventricle/Fontan circulation patients. The first case series published in 2022 by *Muneuchi* et al. included 5 adult Fontan patients who all experienced increased urinary output and reduced edema and/or pleural effusion. No echocardiographic data was evaluated [7]. This was followed by the work of *Konduri* et al., predominantly including pediatric Fontan patients. No formal analysis of the echocardiographic changes was performed, but improvements in global echocardiographic ventricular systolic function were observed in 3 out of 9 patients with sequential measurements available, suggesting that reverse remodeling may be possible [8]. *Skorek* et al. included 17 adult Fontan patients and found a significant improvement in VO_2_ max after starting SGLT2i. They did not observe improvements in S′, TAPSE/MAPSE, E/A ratio, E/e’ ratio, or NT-proBNP. Finally, *Gaydos* et al. recently published a cohort of 25 Fontan patients on SGLT2i, and again found no significant changes in ventricular function [10]. The last two studies both had a mean follow-up of around 11 months and comparisons were only made from baseline to the last follow-up. Our study included a more comprehensive systolic function assessment with repeated assessments per patient and observed significant improvements already in the first 100 days. This could indicate that any beneficial effects of SGLT2i on systolic ventricular function occur early after treatment initiation, which could not be captured by the previous studies.

All four studies concluded that SGLT2i therapy was well-tolerated in the single ventricle population, with cautious signs of beneficial effects [[7], [8], [9], [10]]. These findings are congruent with the first results from the ACHIEVE-SGLT2i registry published in 2024, demonstrating that SGLT2i were safe and well-tolerated in 174 adults with congenital heart disease, of which 12 with single ventricle physiology [11]. Apart from survival analysis showing no differences in the freedom from heart failure hospitalization between systemic ventricular morphology subgroups, no echocardiographic data on the single ventricle cohort was reported in that study [11].

### 4.4 Limitations

This real-world multicenter study is inherently limited by the retrospective design, lack of a control group, and small population size, which reflects the rarity of this type of circulatory failure. The aim was to provide a comprehensive evaluation of longitudinal echocardiographic changes in ventricular function after initiating SGLT2i therapy. Additional statistical analysis of clinical outcome and biomarker data (including natriuretic peptides) was precluded by a paucity of available data. Future research should prioritize correlating biomarkers and imaging data with clinical events to elucidate the potential benefits and mechanisms of action of SGLT2i in single ventricle circulatory failure. Echocardiographic imaging remains challenging in this heterogeneous population. Although high reproducibility of echocardiographic strain and volume measurements has previously been reported in the single ventricle population, intra- or inter-observer variability assessment was not performed in this study [28,29]. Nonetheless, the current study contributes valuable insights to the existing literature by incorporating detailed echocardiographic analysis of sequential TTEs. Mixed models were utilized to correct for individual-level variability, allow the inclusion of unbalanced data with variable follow-up intervals, and provide much-needed evidence for the growing population of single ventricle physiology patients suffering from heart failure.

### 4.5 Conclusions

This study reassuringly demonstrates that SGLT2i therapy is associated with echocardiographic improvements in patients with single ventricle circulatory failure. This includes significant improvements in FWS and basal lateral segmental strain in the first year of treatment, a significant decrease in end-systolic area, and a significant improvement in IVA in both SVFrEF and SVFpEF patients. The observed improvement in FAC specifically in SVFrEF patients suggests that there may be differential responses to SGLT2i therapy between different single ventricle circulatory failure phenotypes. These findings add to the growing evidence for using SGLT2i in single ventricle circulatory failure. Nonetheless, larger comparative studies are imperative to evaluate the response to SGLT2i between different single ventricle circulatory failure phenotypes, study the long-term clinical outcomes, and elucidate the prognostic role of SGLT2i in this complex patient population.

## CRediT authorship contribution statement

**Ralph M.L. Neijenhuis:** Writing – review & editing, Writing – original draft, Visualization, Project administration, Methodology, Investigation, Funding acquisition, Formal analysis, Data curation, Conceptualization. **Madelien V. Regeer:** Writing – review & editing, Supervision, Methodology, Investigation, Conceptualization. **Niki L. Walker:** Writing – review & editing, Resources. **Amanda Hunter:** Writing – review & editing, Resources. **Philippine Kiès:** Writing – review & editing, Resources. **Eduard R. Holman:** Writing – review & editing, Resources. **J. Wouter Jukema:** Writing – review & editing, Supervision. **Monique R.M. Jongbloed:** Writing – review & editing, Supervision, Resources. **Gruschen R. Veldtman:** Writing – review & editing, Supervision, Resources, Methodology, Investigation, Conceptualization. **Anastasia D. Egorova:** Writing – review & editing, Supervision, Resources, Methodology, Investigation, Funding acquisition, Conceptualization.

## Data statement

The data underlying this article will be shared upon reasonable request to the corresponding author.

## Declaration of generative AI in scientific writing

AI and AI-assisted technologies were not used in the writing process of this work. During the preparation of this work the author(s) used ChatGPT 4.0 (OpenAI, San Francisco, CA, USA) for troubleshooting relating to the R script. After using this tool/service, the authors reviewed and edited the content as needed and take full responsibility for the content of the publication.

## Declaration of competing interest

The authors declare the following financial interests/personal relationships which may be considered as potential competing interests: Ralph M.L. Neijenhuis reports financial support was provided by 10.13039/501100001722Royal Netherlands Academy of Arts and Sciences. Ralph M.L. Neijenhuis reports financial support was provided by Foundation De Drie Lichten. Ralph M.L. Neijenhuis reports financial support was provided by AstraZeneca. Anastasia D. Egorova reports financial support was provided by AstraZeneca. Anastasia D. Egorova reports a relationship with Rembrandt Institute for Cardiovascular Sciences that includes: funding grants. Monique R.M. Jongbloed reports a relationship with Rembrandt Institute for Cardiovascular Sciences that includes: funding grants. Monique R.M. Jongbloed reports a relationship with Netherlands Organisation for Health Research and Development that includes: funding grants. Monique R.M. Jongbloed reports a relationship with Bontius Foundation that includes: funding grants. If there are other authors, they declare that they have no known competing financial interests or personal relationships that could have appeared to influence the work reported in this paper, other than Gruschen R. Veldtman serving the Editorial Board of the IJCCHD, but had no involvement with the handling of this paper.
