# Supplementary material for: Echocardiographic effects of sodium-glucose cotransporter 2 inhibitors in single ventricle circulatory failure
Source: Int J Cardiol Congenit Heart Dis. 2025 Jun 21;21:100603. doi: 10.1016/j.ijcchd.2025.100603 (PMC12274904; doi:10.1016/j.ijcchd.2025.100603)
Supplement: Multimedia component 1 [file mmc1.docx]

**SUPPLEMENTARY MATERIAL**

**Title:** Echocardiographic Effects of Sodium-Glucose Cotransporter 2 Inhibitors in Single Ventricle Circulatory Failure

**Contents:**

|  | Page |
| --- | --- |
| **Supplementary Table 1.** Individual anatomical and echocardiographic characteristics at baseline | 2 |
| **Supplementary Table 2.** Mixed models output | 3 |
| **Supplementary Table 3.** Mixed models characteristics | 5 |
| **Supplementary Figure 1.** Changes in TAPSE/MAPSE after starting SGLT2i | 7 |
| Supplementary Figure 1 legend. | 7 |
| **Supplementary Figure 2.** Changes in GLS after starting SGLT2i | 8 |
| Supplementary Figure 2 legend. | 8 |
| **Supplementary Figure 3.** Changes in mid septal strain after starting SGLT2i | 9 |
| Supplementary Figure 3 legend. | 9 |
| **Supplementary Figure 4.** Changes in mid lateral strain after starting SGLT2i | 10 |
| Supplementary Figure 4 legend. | 10 |
| **Supplementary Figure 5.** Individual changes after starting SGLT2i | 11 |
| Supplemetary Figure 5 legend. | 11 |

**SUPPLEMENTARY TABLES**

**Supplementary Table 1.** Individual anatomical and echocardiographic characteristics at baseline

| **Patient** | **Anatomy** | **Global systolic ventricular function** | **GLS (%)** | **FWS (%)** | **FAC (%)** |
| --- | --- | --- | --- | --- | --- |
| **1** | Fontan: LV dominant | Mildly reduced | -17.3 | -17.0 | 29.6 |
| **2** | Other: ccTGA + DORV + VSD + PA | Moderately reduced | -12.1 | -14.0 | 33.0 |
| **3** | Other: DORV + VSD | Moderately reduced | -15.0 | -12.3 | 19.9 |
| **4** | Fonta: RV dominant (HLHS) | Severely reduced | -9.0 | -6.0 | 14.3 |
| **5** | Fontan: mixed/indeterminate ventricle | Severely reduced | -12.8 | -13.3 | 16.9 |
| **6** | Other: DORV + hypoplastic LV cavity + mildly reduced mitral valve orifice + VSD | Moderately reduced | -14.8 | -13.7 | 26.2 |
| **7** | Fontan: RV dominant (HLHS) | Moderately reduced | -11.2 | -11.7 | 23.7 |
| **8** | Fontan: RV dominant (HLHS) | Moderately reduced | -12.0 | -14.0 | 20.5 |
| **9** | Fontan: RV dominant (HLHS) | Mildly reduced | -16.4 | -20.3 | 27.6 |
| **10** | Fontan: LV dominant | Good | NA | NA | 45.2 |
| **11** | Fontan: LV dominant | Severely reduced | -9.3 | -5.7 | 9.8 |
| **12** | Fontan: LV dominant | Good | -18.1 | -16.3 | 25.3 |
| **13** | Fontan: RV dominant (HLHS) | Good | -18.7 | -15.3 | 30.0 |
| *ccTGA, congenitally corrected transposition of the great arteries; DORV, double-outlet right ventricle; FAC, fractional area change; FWS, free wall strain; GLS, global longitudinal strain; HLHS, hypoplastic left heart syndrome; LV, left ventricle; NA, not available; PA, pulmonary atresia; RV, right ventricle; VSD, ventricular septal defect.* | | | | | |

**Supplementary Table 2.** Mixed models output

|  | **Estimate** | **95% CI** | **P-value** |
| --- | --- | --- | --- |
| ***End-diastolic diameter*** |  |  |  |
| Intercept | 60.186 | 52.340 – 69.032 | 0.000 |
| Follow-up time | 0.004 | -0.013 – 0.020 | 0.682 |
| ***Apex-base length*** |  |  |  |
| Intercept | 71.754 | 66.924 – 76.585 | 0.000 |
| Follow-up ≤ 100 days | -0.034 | -0.067 – <-0.001 | 0.062 |
| Follow-up > 100 days | 0.019 | <-0.001 – 0.039 | 0.067 |
| ***Mid diameter*** |  |  |  |
| Intercept | 53.601 | 48.600 – 58.602 | 0.000 |
| Follow-up time | 0.002 | -0.020 – 0.025 | 0.846 |
| ***Free wall thickness*** |  |  |  |
| Intercept | 9.936 | 8.863 – 11.010 | 0.000 |
| Follow-up time | -0.003 | -0.007 – <0.001 | 0.087 |
| ***End-diastolic area*** |  |  |  |
| Intercept | 35.759 | 30.711 – 40.808 | 0.000 |
| Follow-up ≤ 100 days | -0.023 | -0.067 – 0.021 | 0.323 |
| Follow-up > 100 days | 0.021 | -0.001 – 0.043 | 0.071 |
| ***End-systolic area*** |  |  |  |
| Intercept | 24.009 | 19.627 – 28.391 | 0.000 |
| Follow-up ≤ 100 days | -0.053 | -0.087 – -0.019 | **0.007** |
| Follow-up > 100 days | 0.018 | 0.001 – 0.036 | 0.051 |
| ***FAC*** |  |  |  |
| Intercept | 32.353 | 26.642 – 38.064 | 0.000 |
| Follow-up ≤ 100 days | 0.114 | 0.065 – 0.162 | **<0.001** |
| Follow-up > 100 days | -0.020 | -0.045 – 0.005 | 0.141 |
| SVF phenotype = SVFpEF | -1.096 | -10.207 – 8.015 | 0.818 |
| Interaction SVFpEF and follow-up ≤ 100 days | -0.117 | -0.192 – -0.042 | **0.007** |
| Interaction SVFpEF and follow-up > 100 days | 0.050 | -0.003 – 0.102 | 0.081 |
| ***TAPSE/MAPSE*** |  |  |  |
| Intercept | 10.299 | 9.186 – 11.411 | 0.000 |
| Follow-up time | 0.001 | -0.002 – 0.005 | 0.515 |
| SVF phenotype = SVFpEF | 2.708 | 1.004 – 4.412 | **0.010** |
| ***GLS*** |  |  |  |
| Intercept | -12.220 | -13.153 – -11.288 | 0.000 |
| Follow-up time | -0.004 | -0.008 – <0.001 | 0.087 |
| SVF phenotype = SVFpEF | -5.348 | -6.334 – -4.362 | **<0.001** |
| ***FWS*** |  |  |  |
| Intercept | -11.697 | -13.346 – -10.047 | 0.000 |
| Follow-up time | -0.008 | -0.016 – -0.001 | **0.036** |
| SVF phenotype = SVFpEF | -4.721 | -7.121 – -2.322 | **0.003** |
| ***Basal septal segmental strain*** |  |  |  |
| Intercept | -10.263 | -14.852 – -5.675 | <0.001 |
| Follow-up time | -0.010 | -0.021 – 0.002 | 0.120 |
| ***Mid septal segmental strain*** |  |  |  |
| Intercept | -13.126 | -16.803 – -9.450 | 0.000 |
| Follow-up time | -0.006 | -0.018 – 0.007 | 0.394 |
| SVF phenotype = SVFpEF | -5.022 | -8.577 – -1.468 | **0.018** |
| ***Apical septal segmental strain*** |  |  |  |
| Intercept | -16.580 | -18.909 – -14.521 | 0.000 |
| Follow-up time | -0.008 | -0.019 – 0.003 | 0.154 |
| ***Apical lateral segmental strain*** |  |  |  |
| Intercept | -13.365 | -15.328 – -11.402 | 0.000 |
| Follow-up time | -0.009 | -0.021 – 0.002 | 0.120 |
| ***Mid lateral segmental strain*** |  |  |  |
| Intercept | -11.638 | -14.102 – -9.175 | 0.000 |
| Follow-up time | -0.003 | -0.011 – 0.005 | 0.487 |
| SVF phenotype = SVFpEF | -4.916 | -8.676 – -1.157 | **0.026** |
| ***Basal lateral segmental strain*** |  |  |  |
| Intercept | -10.644 | -13.459 – -7.829 | 0.000 |
| Follow-up time | -0.010 | -0.019 – -0.002 | **0.032** |
| SVF phenotype = SVFpEF | -7.630 | -11.968 – -3.292 | **0.006** |
| ***IVA*** |  |  |  |
| Intercept | 3.281 | 2.406 – 4.157 | <0.001 |
| Follow-up ≤ 100 days | 0.018 | 0.007 – 0.028 | **0.010** |
| Follow-up > 100 days | -0.006 | -0.012 – -<0.001 | 0.075 |
| SVF phenotype = SVFpEF | 1.250 | 0.341 – 2.159 | **0.027** |
| ***S’*** |  |  |  |
| Intercept | 0.070 | 0.057 – 0.083 | 0.000 |
| Follow-up time | <-0.001 | <-0.001 – <0.001 | 0.889 |
| ***E/A ratio*** |  |  |  |
| Intercept | 1.616 | 1.280 – 1.951 | 0.000 |
| Follow-up time | <-0.001 | -0.001 – 0.001 | 0.626 |
| ***E/e’ ratio*** |  |  |  |
| Intercept | 7.289 | 4.864 – 9.714 | <0.001 |
| Follow-up time | 0.005 | -0.003 – 0.013 | 0.289 |
| Estimates are displayed as absolute intercept values or temporal coefficients of the predicted changes per day. *FAC, fractional area change; FWS, free wall strain; GLS, global longitudinal strain; IVA, isovolumic acceleration; SVF, single ventricle failure; SVFrEF/SVFpEF, single ventricle failure with reduced/preserved ejection fraction; TAPSE/MAPSE, tricuspid/mitral annular plane systolic excursion.* | | | |

**Supplemental Table 3.** Mixed models characteristics

|  | **Time relation** | **Correlation structure** | **Variance structure** | **Covariate** |
| --- | --- | --- | --- | --- |
| End-diastolic diameter | Linear model with random intercepts | Unspecified | Unspecified | NS |
| Apex-base length | Piecewise linear model with random intercepts and a knot at 100 days | Unspecified | Unspecified | NS |
| Mid diameter | Linear model with random intercepts and random slopes | Unspecified | Unspecified | NS |
| Free wall thickness | Linear model with random intercepts | Unspecified | Unspecified | NS |
| End-diastolic area | Piecewise linear model with random intercepts and a knot at 100 days | Unspecified | VarPower | NS |
| End-systolic area | Piecewise linear model with random intercepts and a knot at 100 days | Unspecified | VarExp | NS |
| FAC | Piecewise linear model with random intercepts and a knot at 100 days | Unspecified | Unspecified | Significant interaction between SVF phenotype and time before 100 days. No significant fixed effect. |
| TAPSE/MAPSE | Linear model with random intercepts | Unspecified | Unspecified | Fixed effect for SVF phenotype. |
| GLS | Linear model with random intercepts | Unspecified | VarPower | Fixed effect for SVF phenotype. |
| FWS | Linear model with random intercepts | Unspecified | Unspecified | Fixed effect for SVF phenotype. |
| Basal septal segmental strain | Linear model with random intercepts | Unspecified | Unspecified | NS |
| Mid septal segmental strain | Linear model with random intercepts and random slopes | Unspecified | Unspecified | Fixed effect for SVF phenotype. |
| Apical septal segmental strain | Linear model with random intercepts | Unspecified | Unspecified | NS |
| Apical lateral segmental strain | Linear model with random intercepts | Unspecified | Unspecified | NS |
| Mid lateral segmental strain | Linear model with random intercepts | Unspecified | Unspecified | Fixed effect for SVF phenotype. |
| Basal lateral segmental strain | Linear model with random intercepts | Unspecified | Unspecified | Fixed effect for SVF phenotype. |
| IVA | Piecewise linear model with random intercepts and a knot at 100 days | Unspecified | Unspecified | Fixed effect for SVF phenotype. |
| S’ | Linear model with random intercepts | Unspecified | Unspecified | NS |
| E/A ratio | Linear model with random intercepts | Unspecified | Unspecified | NS |
| E/e’ ratio | Linear model with random intercepts | Unspecified | Unspecified | NS |
| *FAC, fractional area change; FWS, free wall strain; GLS, global longitudinal strain; IVA, isovolumic acceleration; NS, not significant; SVF, single ventricle failure; TAPSE/MAPSE, tricuspid/mitral annular plane systolic excursion.* | | | | |

**SUPPLEMENTARY FIGURES**

**Supplementary Figure 1.** Changes in TAPSE/MAPSE after starting SGLT2i


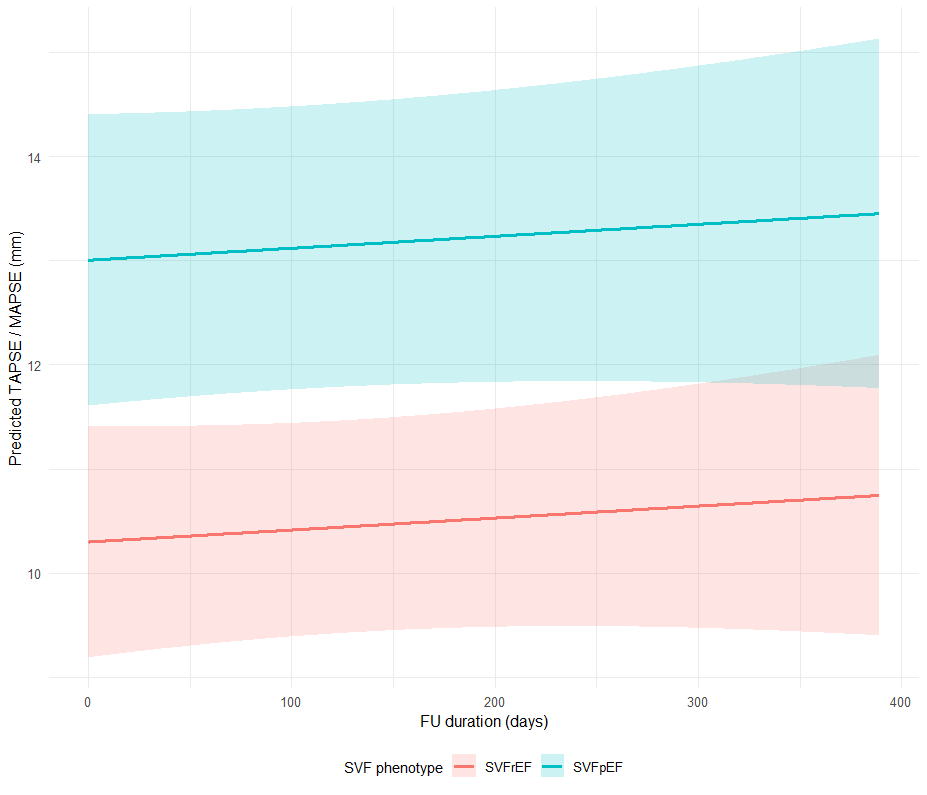


**Supplementary Figure 1 Legend.** Predicted values of TAPSE/MAPSE over time with 95% confidence interval, faceted for the SVF phenotypes. *FU, follow-up; SGLT2i, sodium-glucose cotransporter 2 inhibitor; SVF, single ventricle failure; SVFrEF/SVFpEF, single ventricle failure with reduced/preserved ejection fraction; TAPSE/MAPSE, tricuspid/mitral annular plane systolic excursion.*

**Supplementary Figure 2.** Changes in GLS after starting SGLT2i


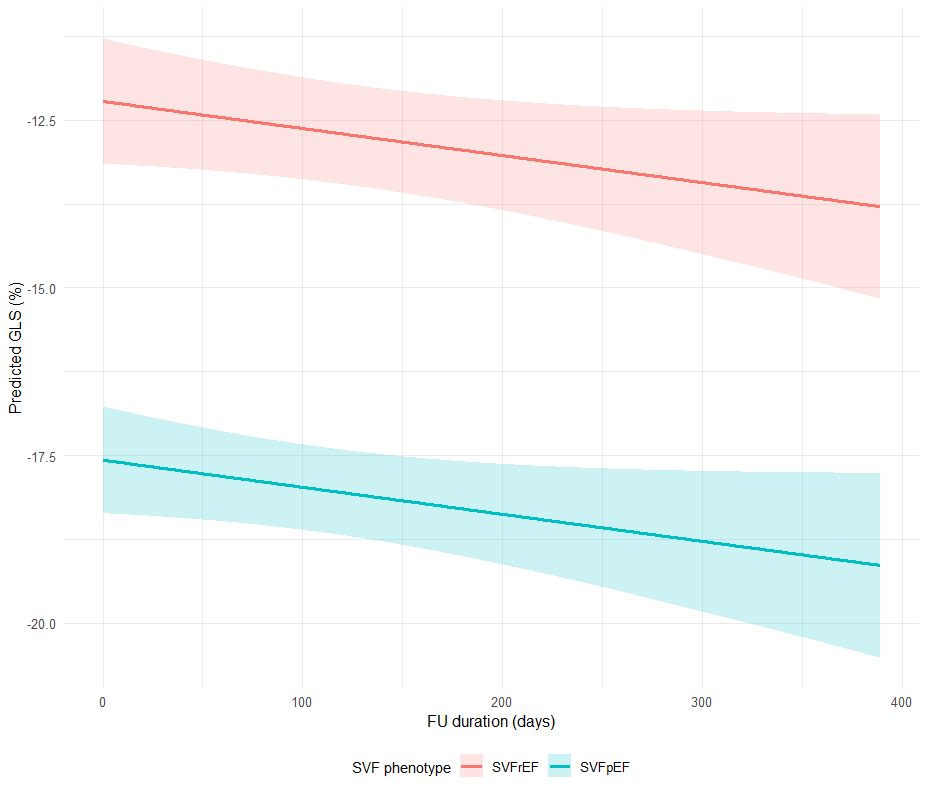


**Supplementary Figure 2 Legend.** Predicted values of GLS over time with 95% confidence interval, faceted for the SVF phenotypes. *FU, follow-up; GLS, global longitudinal change; SGLT2i, sodium-glucose cotransporter 2 inhibitor; SVF, single ventricle failure, SVFrEF/SVFpEF, single ventricle failure with reduced/preserved ejection fraction.*

**Supplementary Figure 3.** Changes in mid septal strain after starting SGLT2i


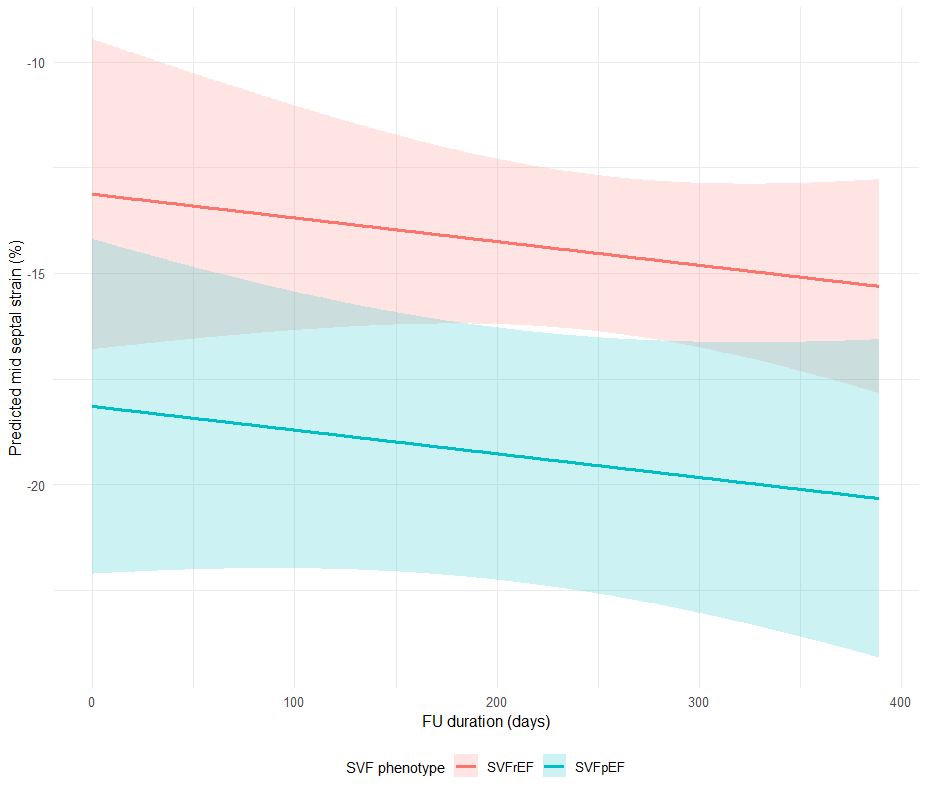


**Supplementary Figure 3 Legend.** Predicted values of mid septal strain over time with 95% confidence interval, faceted for the SVF phenotypes. *FU, follow-up; SGLT2i, sodium-glucose cotransporter 2 inhibitor; SVF, single ventricle failure; SVFrEF/SVFpEF, single ventricle failure with reduced/preserved ejection fraction.*

**Supplementary Figure 4.** Changes in mid lateral strain after starting SGLT2i


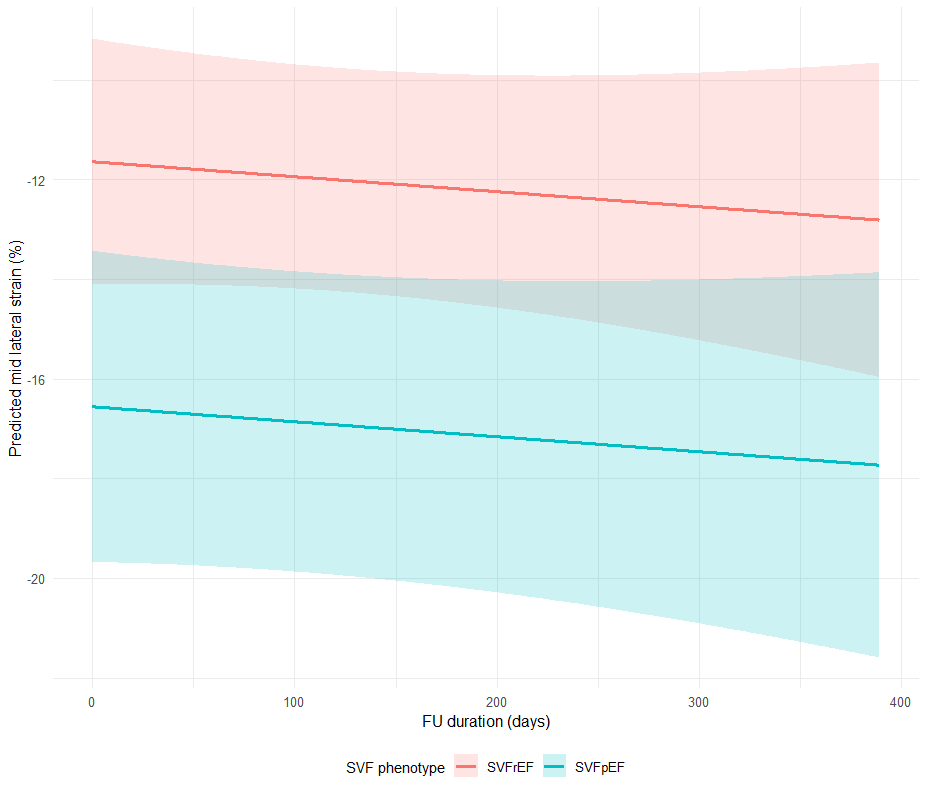


**Supplementary Figure 4 Legend.** Predicted values of mid lateral strain over time with 95% confidence interval, faceted for the SVF phenotypes. *FU, follow-up; SGLT2i, sodium-glucose cotransporter 2 inhibitor; SVF, single ventricle failure; SVFrEF/SVFpEF, single ventricle failure with reduced/preserved ejection fraction.*

**Supplementary Figure 5.** Individual changes after starting SGLT2i


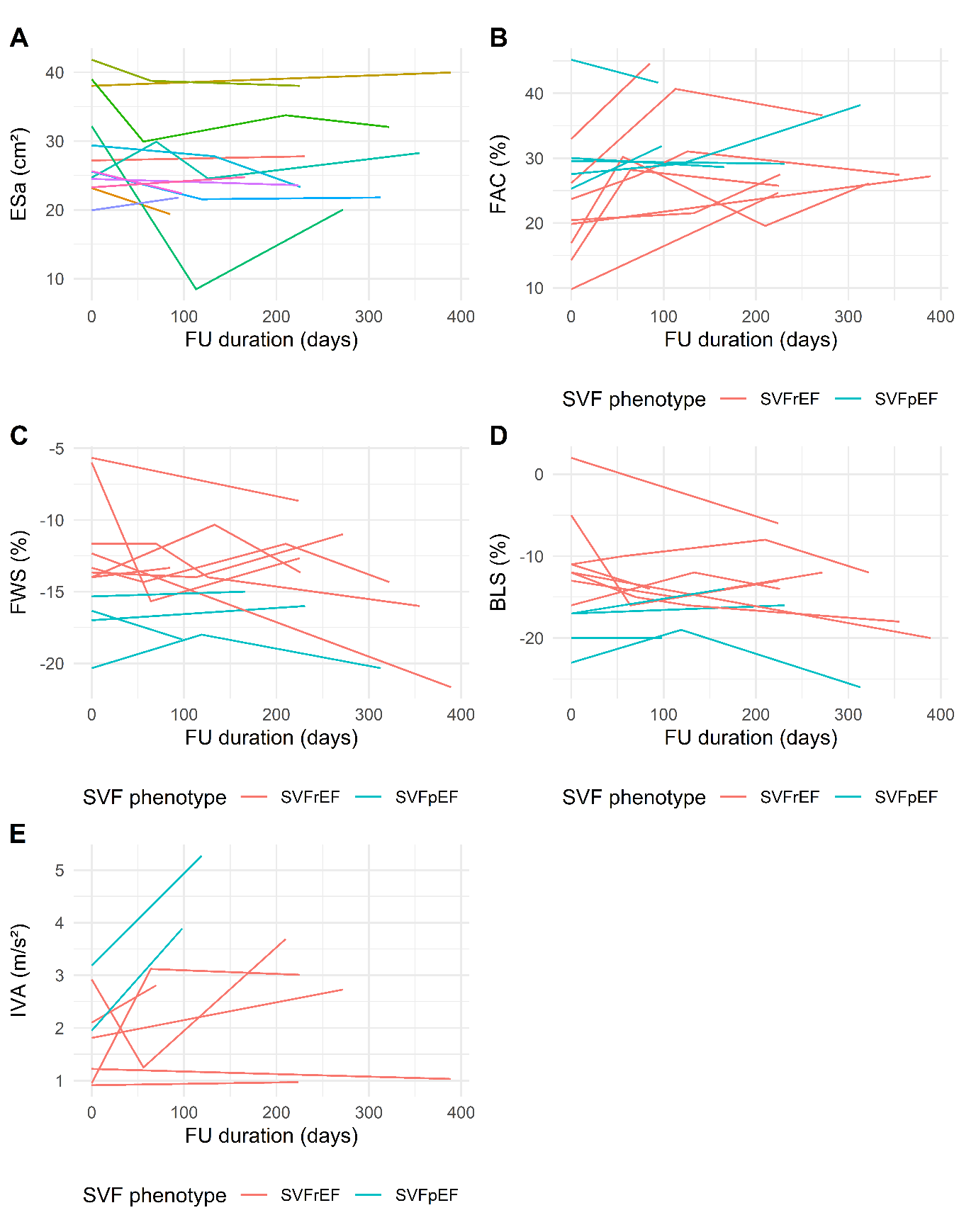


**Supplementary Figure 5 Legend.** Individual changes for parameters with significant temporal changes after starting SGLT2i. The lines are stratified by SVF phenotype when this was a significant covariate in the corresponding mixed model. *BLS, basal lateral strain; Esa, end-systolic area; FAC, fractional area change; FU, follow-up; FWS, free wall strain; IVA, isovolumic acceleration; SGLT2i, sodium-glucose cotransporter 2 inhibitor; SVF, single ventricle failure; SVFrEF/SVFpEF, single ventricle failure with reduced/preserved ejection fraction.*
